# Current Perspectives of Artificial Intelligence in Pediatric Neuroradiology: An Overview

**DOI:** 10.3389/fradi.2021.713681

**Published:** 2021-09-07

**Authors:** Dann Martin, Elizabeth Tong, Brendan Kelly, Kristen Yeom, Vivek Yedavalli

**Affiliations:** ^1^Vanderbilt University, Nashville, TN, United States; ^2^Department of Neuroradiology, Stanford Health Care, Stanford, CA, United States; ^3^Insight Centre for Data Analytics, University College Dublin, Dublin, Ireland; ^4^Johns Hopkins University, Baltimore, MD, United States

**Keywords:** neuroradiology, artificial intelligence, machine learning, pediatric neuroradiology, deep learning

## Abstract

Artificial Intelligence, Machine Learning, and myriad related techniques are becoming ever more commonplace throughout industry and society, and radiology is by no means an exception. It is essential for every radiologists of every subspecialty to gain familiarity and confidence with these techniques as they become increasingly incorporated into the routine practice in both academic and private practice settings. In this article, we provide a brief review of several definitions and techniques that are commonly used in AI, and in particular machine vision, and examples of how they are currently being applied to the setting of clinical neuroradiology. We then review the unique challenges that the adoption and application of faces within the subspecialty of pediatric neuroradiology, and how these obstacles may be overcome. We conclude by presenting specific examples of how AI is currently being applied within the field of pediatric neuroradiology and the potential opportunities that are available for future applications.

## Introduction

Advances in artificial intelligence (AI) could fundamentally change how millions of people will live and work. Medicine is one field that is particularly amenable to the potential everyday impacts of AI. Due to some exciting advances within the last decade within the field of machine vision, medical specialties that rely on image analysis are particularly susceptible to such revolution; disciplines such as radiology, pathology, dermatology, and ophthalmology are among those that are actively preparing to incorporate AI as a part of daily workflows (1, 2).

AI is a branch of computer science that focuses on developing complex functions or “algorithms” that may be used as in determining the solution to an arbitrary desired problem. While AI has its theoretical origins as far back as the 1950's, the field has experienced something of a renaissance with the past few decades due to the recent exponential increase in computational power coupled with decreased cost as well as increased availability of the type of computer processors necessary to accomplish the huge amount of computation necessary for training AI systems. AI is an overarching term that comprises a variety of techniques used to achieve the ultimate task of determining the solution to an arbitrary problem. Within the context of medicine, and specifically radiology, the most relevant subfields of AI are neural networks, deep learning (DL), and machine vision (1, 3, 4).

In this review article, we start with a general description of AI techniques commonly used in neuroradiology. This is followed by a few examples of AI in Pediatric Neuroradiology. We will highlight a number of factors that may contribute to the relative disparity between AI's deployment in adult and pediatric radiology. Finally, we will discuss why pediatric radiology is, in fact, the ideal setting for a number of specific deployments of medical AI. Our objective of this review is that it provides an overview of the increasingly prevalent and essential concepts of AI's role in neuroradiology. With a minimal amount of education of the field (and this review indeed only scratches the surface of the subject), the anxiety that many radiologists feel when approaching the mysterious and apparently daunting concepts of AI may dissipate and be replaced with a genuine curiosity and even a sense of wonder regarding the power and potential of such a revolutionary technology. Furthermore, after the basic techniques have been introduced, we hope to show how deeply ingrained the technology has already become in some areas of neuroradiology practice as well as how much potential still exists in others, notably in pediatric neuroradiology.

### Impact of AI Applications

Given the remarkable success of AI and its potential applications within radiology, interest has grown tremendously, but, with these advances, come additional responsibilities that significantly affect practicing radiologists in their daily workflow. Radiology is more and more relied upon as an essential non-invasive diagnostic tool that is crucial in the guiding medical decision making and monitoring. Because of this, there has been a continuous increase in volume of radiologic studies over the past few decades, while the number of active radiologists responsible for their interpretation has remained relatively stable or even decreased (5). In addition to the increase in volume of imaging, the complexity of the technology with which we are able to image is always increasing as well, meaning more tools are available in our diagnostic arsenal. Unfortunately, these advanced techniques are typically additive rather than alternative, and the amount of imaging that is done per study, and therefore number of images a radiologist is responsible for, also tends to increase over time. To make matters worse, technology is also always improving efficiency of acquiring and processing studies, meaning more imaging can be done in less time. These issues coupled together mean that there is an ever-growing number of studies, all becoming more complex, and all coming at radiologists faster than ever before. While this may be favorable for the bottom line of the radiology department, the sentiment is rarely shared by the increasingly exhausted and fatigued staff, and physician burnout has become a serious issue felt by both staff and patients (6).

AI presents a potential solution to this growing problem. If a neural network can be trained to recognize patterns and lesions as well as their human counterparts, they would be able to convert a set of images into a report on the order of milliseconds, where it may take the most efficient radiologist several minutes to perform such a task well (7). This means that an entire department's daily volume could be completed by an AI system in a matter of seconds, and a department's throughput would be limited only by how fast it could pass patients through the scanners, a task that itself is becoming more rapid thanks to machine learning techniques (8).

Although in its infancy, applications of machine learning in radiology have already had a large impact. Perhaps most famously, algorithms trained to read chest X-rays with near-human accuracy have been widely publicized, though have also faced some criticism (9–11). Within the subspecialty of neuroradiology, many algorithms have been or are being developed for the purposes of automated segmentation, aneurysm detection, and stroke diagnosis and prognosis, among many others (3, 12–26). Much less common are applications of AI into pediatric radiology and, even more rare, pediatric neuroradiology.

### Neural Network

**Figure 1:**
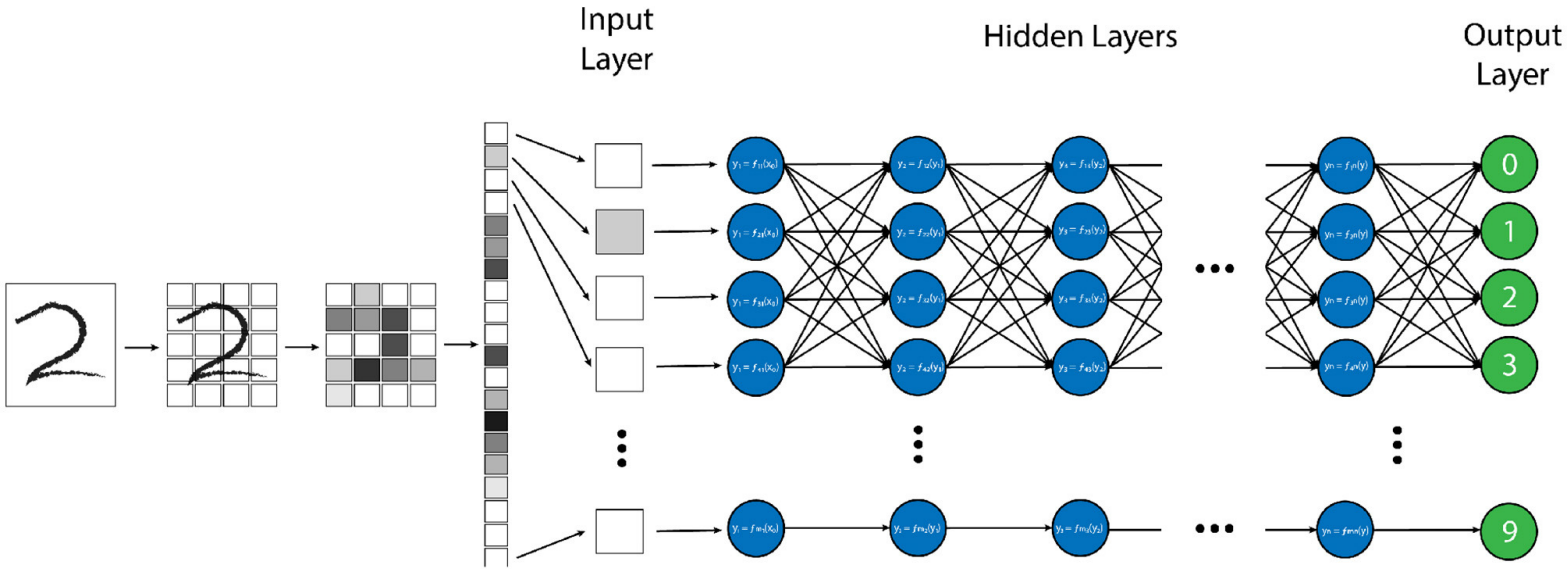
Example of a “vanilla” neural network, designed for handwritten number recognition. In this algorithm, a handwritten figure is pixelated into shades of gray, and these pixels are vectorized as an input into the first layer of the neural network. Each node (blue circle) performs a simple mathematical transform of the input data (in this case the grayscale pixel value) and passes that transformed value as an output to each node in the next layer. Each node in the subsequent layer will create a weighted average of all of the outputs from the prior layer as its input and perform another simple transform on this value. This process is repeated through each layer of the network until the final “output” layer (green circles) is reached, which, in this model, will decide which of the 10 possible numbers the provided image is most similar to.

There are many strategies and computational structures that AI uses to accomplish its task. One of the most successful techniques has been neural networks (NN) (1–4). NNs are a system of data processing in which an arbitrary type of data (e.g. pixel values from an image, letters or symbols from text, or waveforms from an audio recording) is input into the system in the form of an ordered array of values, known as an “input vector” (3). This input vector is then fed into the first “layer” network, where mathematical transforms are applied to the components of the vector, which augment the data in some non-linear way. The components of this transformed vector are then combined into a weighted sum, which redistributes the data into a new “output vector”, which itself is then passed to the next layer of the network where this entire process is repeated (1, 3). By repeating this process through an arbitrary number of layers, the original input vector is transformed to an ultimate output vector with values that correspond to some desired output data distribution for a given input vector, such as the probability distribution that a certain vector of pixel values corresponds to a specific element in list of objects (Figure 1) (3).

**Figure 2:**
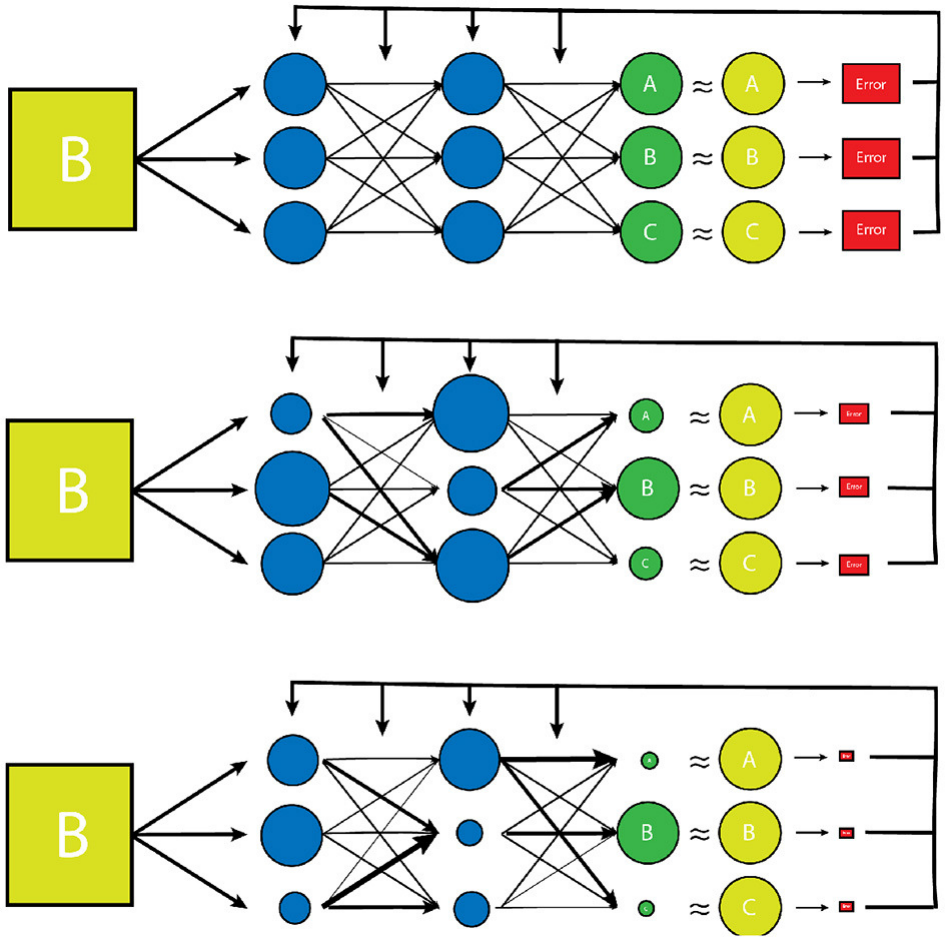
A simplified model of the training process of a vanilla neural network. Initially, the transform parameters and weights associated with the network are completely random. During training of the network, inputs paired with predetermined desired outputs (the training set) are run through the network and an error is determined by comparing how close the network's output distribution came to the desired “gold standard” output (yellow circles). The network parameters and weights are then readjusted slightly (represented by varying node size and arrow thickness) and the input is tested again. The new error is then compared to the prior, and the network parameters are adjusted in such a way that error continues to decrease with each testing iteration. Once the error has reached a desired minimum range, a new example from the training set is used to further reduce the error of the network. Once all of the training set examples have been evaluated and a desired error has been achieved, the network is considered “trained”. This trained network is then tested on a smaller set of known input/output examples (test set) to ensure that the model can work well on examples that it has never seen before.

There are a large number of variables and weights in the NN that are optimized to produce the desired output. The network is designed to adjust these variables independently until it has “learned” the parameters such that the final output data has the desired “correct” result for a given input, determined by minimizing a predesignated error function (Figure 2) (27). The error function is another independent parameter of the algorithm and may be determined by the human dictating which output vector are “correct”, or the “ground truth” in a process known as supervised learning which typically involves the arduous task of manually labeling large datasets. Alternatively, the network can learn to detect patterns in its dataset itself, and create its own definition of “correct”, based on how it categorizes its input dataset into groups of some kind of inherent similarity, which is known as unsupervised learning. Regardless of how a network determines the error of its output data, this “training” process is repeated many times for each example within a dataset until the variables have been selected that gives the results with the smallest amount of error (1, 2, 28, 29). The space of potential parameter values is enormous for most tasks, and even the fastest available processors typically take days or even weeks to independently optimize the millions of variables, making the process extremely costly both in terms of time and necessary equipment (29).

Beyond the intrinsic variables within a neural network that must be optimized, there are many additional “hyperparameters” that can be adjusted, so that the network trains as efficiently and thoroughly as possible. Among such hyperparameters is the structure of the network itself. Most traditional networks function in layers, which represents a single step in the algorithm where input data is transformed, mixed together, then passed onto the next layer. The layer system generally allows data to be represented in increasingly abstract ways as it progresses through the network. For example, if the input layer for a network is the gray-level pixel data from a black and white image of a person's face, each unit in the first layer would contain a value which represents a single pixel of that image. This data would then be adjusted and combined in some way and passed onto the next layer, where each unit of which may now represent a combination of pixels that form certain patterns such as lines or curves. These data would then themselves be mixed together and passed to the next layer where the lines and curves would be combined into patterns such as circles or triangles. This process would be repeated until the units represented something increasingly abstract such as an eyebrow, the curve of a cheekbone, or a smiling mouth. Ultimately, these facial features would be combined in an output layer which would label the image as a “face”, possibly out of list of potentially thousands of objects. The more layers that are available for a network to compute with and increase the abstraction of the data, the “deeper” the network is said to be. While a deeper network is not necessarily always superior in machine learning, it often helps in making the network more robust, and such “deep learning” algorithms have become a crucial component of the success of modern AI. The types of layered networks described thus far are typically referred to as “vanilla” neural networks, in that they are the simplest type of network architecture that is routinely used and deployed. Many additional types architectural complexity may be added to these simpler vanilla NNs including techniques such as recurrence and convolution which increase the complexity of the algorithm, but can be very well suited to specific learning tasks such as natural language processing or image classification.

### Machine Vision

**Figure 3:**
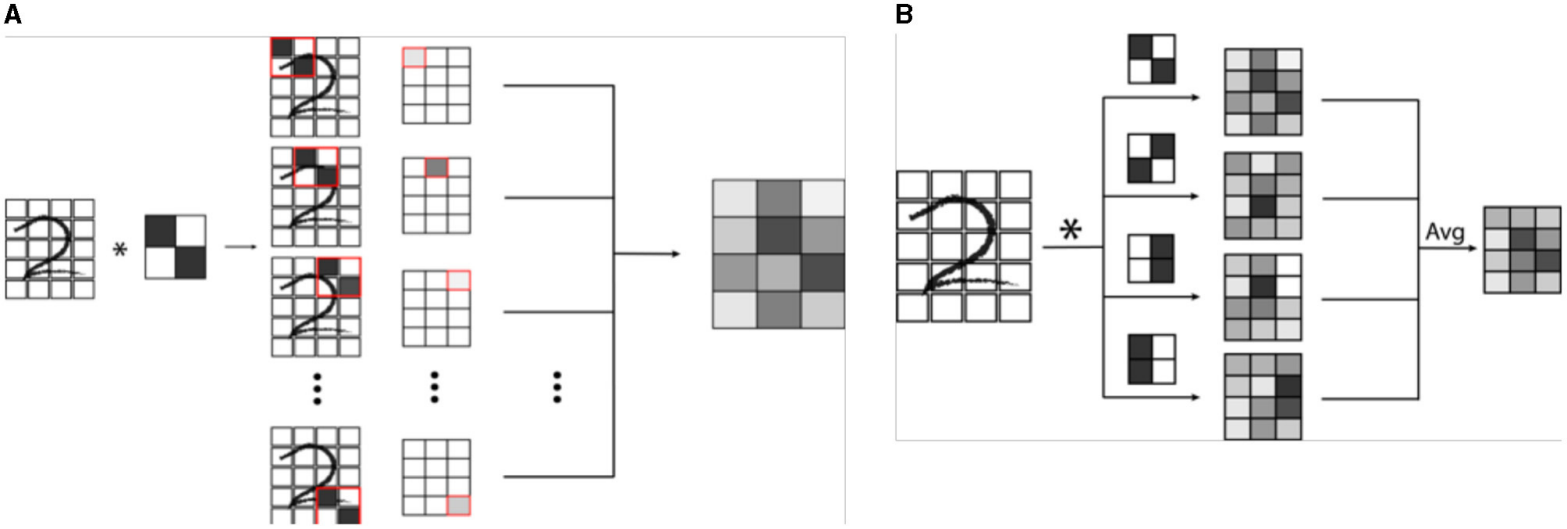
**(A)** Example of a single convolution. A convolutional neural network (CNN) is a specialized network designed for image recognition. A convolution involves the use a mask or filter pattern called a “kernel” that essentially “scans” an input image, and outputs a map of how similar each part of the input image is to the filter. In the example, a simple 2 x 2 pixel kernel with a diagonal line is convolved (denoted by ^*^) with a handwritten digit. As the kernel is passed over each set of 2 x 2 pixels of the image, a grayscale value is generated which is proportional to how well the kernel image “matches” that section of the image. **(B)** In a CNN, many kernels are passed over the same image to create a series of convolutions, which are combined in a weighted average to an output convoluted image. This image is then itself iteratively convolved a number of times, ultimately creating an image that is encoded by all of the convolutional kernels.

**Figure 4:**
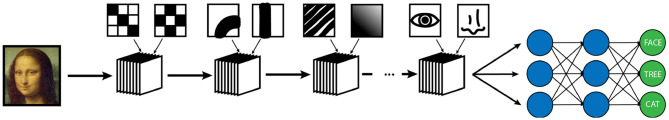
Example of a simplified CNN designed for object recognition/classification. A series of convolutional masks (represented as a stack of elements) are applied to an input image to create increasingly abstract representations of the initial image. At each step of the network, the output is convolved with masks of increasing complexity to classify image features of increasing abstraction. In this example, the first layer of convolutions is measuring simple low level pixel patterns, then larger features such as lines and curves, then textural patterns such as ridges or gradients, and finally larger scale features such as eyes or noses. Following the convolutional encoding process, the output is then fed into a traditional neural network, which is then used to classify the image into a set number of outputs. The weights of convolution averaging as well as the parameters of the classification network are adjusted during the training period in the same way as a traditional neural network.

Machine vision is a specific subtype of AI which focuses on developing algorithms capable of identifying objects and contexts within images, as our own visual cortex is capable of doing (2, 4, 28). Enabling computer systems to be able to see the world around them would fundamentally change how they are able to interact with the world. From self-driving cars to facial recognition, we are currently seeing how powerful and pervasive this developing technology is becoming (28). Machine vision is also perhaps the most important deployment of AI in medicine, and certainly in radiology (2, 29). Modern machine vision utilizes a specialized type of neural network known as a convolutional neural network or CNN (3). CNN's are distinct from typical vanilla neural networks in that they use small images typically consisting of only a few pixels called “kernels” to scan an image, and generate a value depending on a multiplication rule between the kernel and the part of the image being scanned, a process known as “convolution” (Figure 3) (30–33). The data that is generated is then transformed and combined, similarly to a vanilla network, which then forms another image that is smaller, and usually more abstract. This process is repeated with a number of different kernels, and is iterated until a much smaller, more abstract image is obtained (30). This image is typically then run through a vanilla deep neural network, which then classifies it into an arbitrary number of categories (Figure 4). The network trains itself to optimize the parameters, as in vanilla networks, but now also determines the kernels used to convolve the image, as well as the parameters which determine how they these kernels will interact (3). While more complicated, CNNs have shown an amazing advantage for categorizing images over traditional neural networks and have become the gold standard for machine vision tasks, including in medical AI (33).

## Applications of AI in Pediatric Neuroradiology

### Dose/Exam Time Reduction

One deployment of AI in neuroimaging that would disproportionately benefit the pediatric population is in reducing the time of image acquisition. As any radiologist who has had to perform a scan check or conduct a study under fluoroscopy knows, acquiring decent imaging on a squirming infant or screaming toddler can be challenging. Even more than this, obtaining images of diagnostic quality while minimizing radiation dose and scan time is one of the biggest challenges in pediatric radiology. The widespread Image Gently and Step Lightly campaigns from ACR are examples which emphasize the importance of this concept. As previously discussed in adult imaging, current advances in machine learning are displaying a lot of promise in potentially aiding in these goals (34).

**Figure 5:**
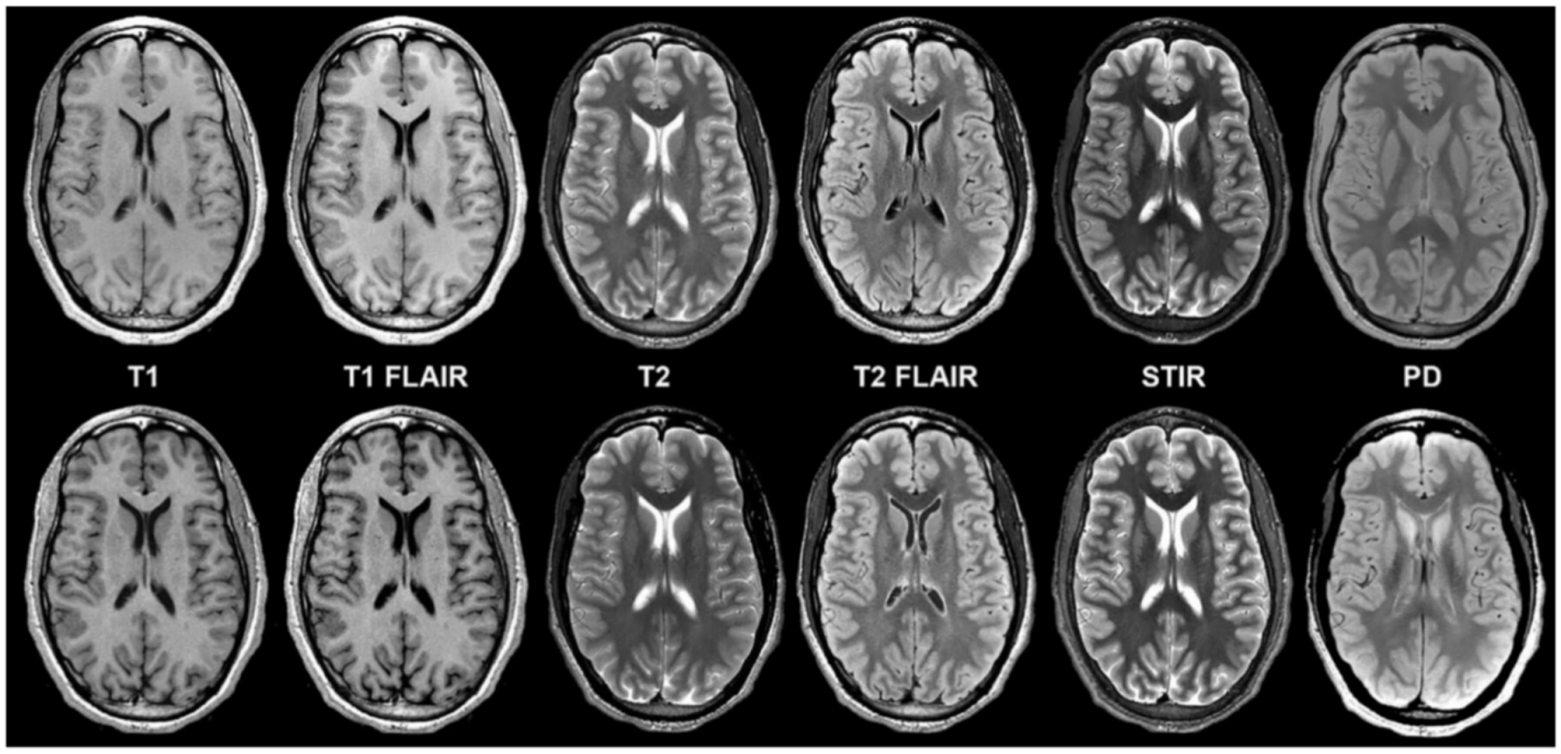
Example of a “MAGiC” sequence of synthetic MRI, in which a standardized set of sequences is constructed from a single scan, the total time of acquisition being about 5 minutes. The sequences in the upper row are conventionally acquired, while those in the lower row are synthetic (24). Reprinted from Tanenbaum et al. (36).

The development of Generative Adversarial Networks (GANs) and algorithms capable of interpolating data from other sequences has shown the ability to significantly increase SNR in images, making previously non-diagnostic fast imaging techniques now suitable for interpretation (35). In the adult field, there has been a push to develop extremely short protocols for emergent indications, using a neural network to interpolate data in a technique known as “Synthetic MRI” (36–39). It is currently possible to obtain an array of sequences from very noisy rapidly acquired single sequence and reconstruct this data into an array of 6 basic sequences with acquisition times of less than 5 minutes, using techniques known as Magnetic Resonance Image Compilation or “MAGiC”, or even faster using ultrafast EPI sequences to produce the “EPImix” series in less than a minute (8, 36) (Figure 5). Application of AI techniques such as GANs to such ultrafast techniques could aid in further improving their SNR, resulting in increased image quality or even faster acquisition. Such short sequences could enable the acquisition of necessary imaging in patients who are unable or unwilling to remain still for the duration of a normal study, particularly in patients with developmental delay or claustrophobia who could otherwise not participate in a full exam. In many cases this could reduce the necessity of sedating or intubating patients for whom the studies are clinically required, which is of obvious benefit to both the patient and provider.

**Figure 6:**
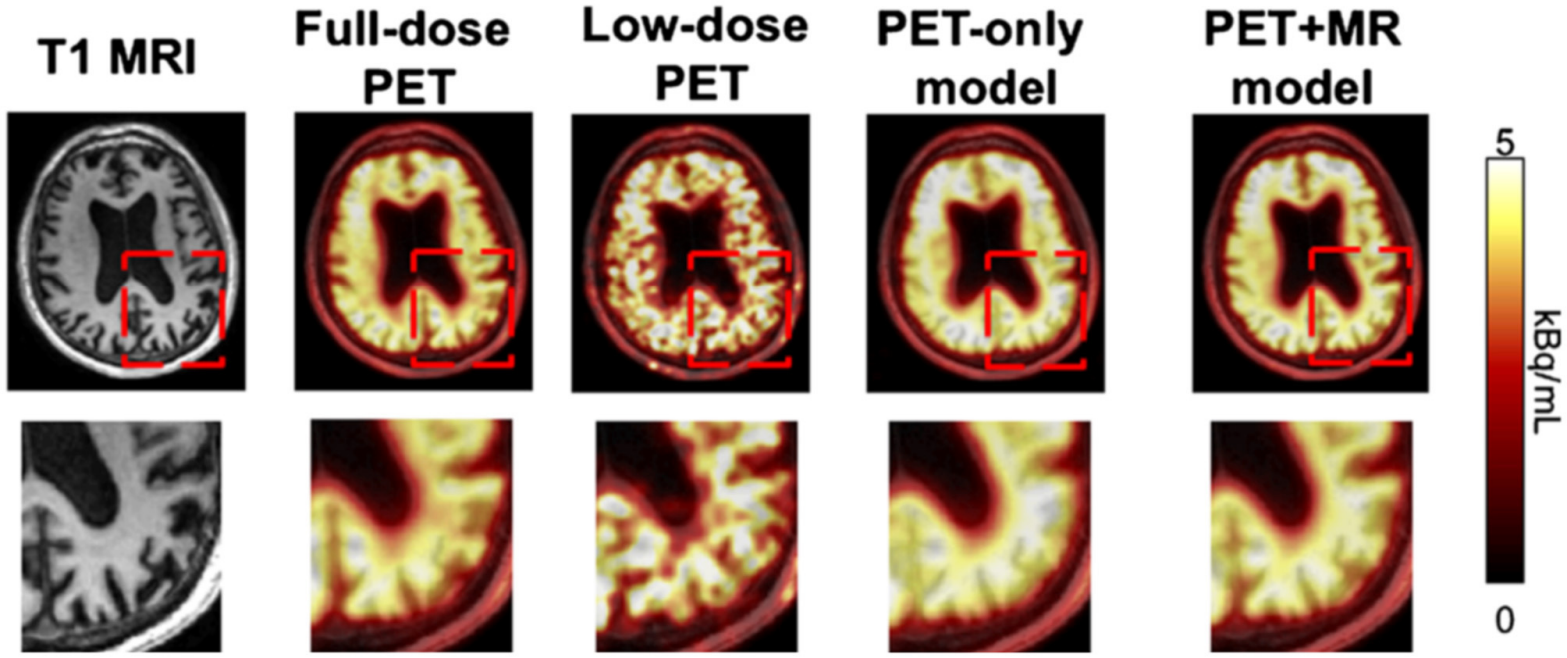
By training a CNN on simultaneously acquired MRI and PET data from PET/MRI, it is possible to generate an algorithm which can interpolate data in a low resolution low dose PET scan to generate more accurate imaging than would be produced by that same algorithm trained on only the PET data (22). Reprinted from Zaharchuk (40) with permission under the terms of the Creative Commons Attribution License, which permits unrestricted use, distribution, and reproduction in any medium, provided the original author and source are credited.

To push the limitations of imaging even further, it has been shown that it is possible to interpolate data from one modality using a neural network trained on data from a completely different modality, for example, improving the resolution of low dose PET imaging by training a neural network with simultaneously acquired PET/MRI images (40) (Figure 6). While the pediatric imaging is moving more and more toward the radiation free modalities of MRI and ultrasound, in situations where CT or PET are required, replacing PET/CT with MRI while still providing vital diagnostic information, can eliminate radiation exposure, which is particularly beneficial and desired in the pediatric population.

### QI/QA

**Figure 7:**
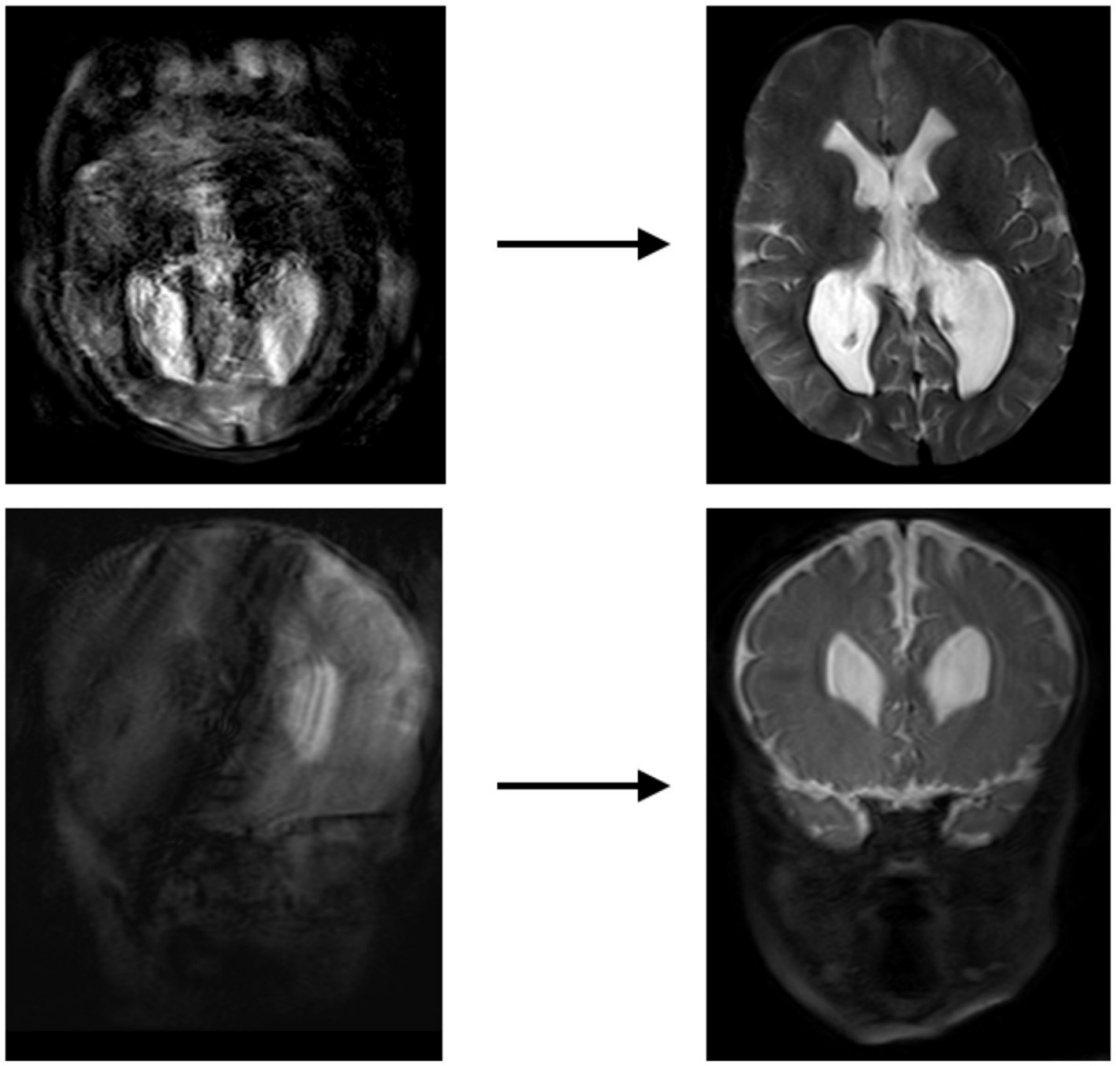
Examples of how badly motion can degrade pediatric imaging. Even taking advantage of rapid protocol MRIs, pediatric patients are notorious for moving during the exam, making non-diagnostic imaging a frustrating and costly challenge for pediatric neuroradiologists. Often, if imaging is absolutely necessary, the patient must undergo general anesthesia to acquire images of diagnostic quality, which comes with its own disadvantages. AI could play a useful role in radiology by creating synthetic images that either artificially create non degraded images based on prior training of matched motion degraded and non-degraded images or by manipulating acquired data within k-space, and generating motion restricted images.

In addition to reducing the dose and time required to obtain imaging, ensuring that scans are of acceptable quality and uniformity is also important. There has been a large push by the ACR and other organizations to emphasize and improve the quality and efficiency of imaging (41). As discussed above, many attempts at reducing the time required to acquire a study. While decreasing the time to complete a study is useful on its own, it can also be used to improve the quality of an image by allowing more acquisitions to be averaged over, which can significantly reduce SNR (Figure 7). Such improvements would ensure that the acquired imaging is as high-quality as possible, while simultaneously reducing the need for patients to return for technically inadequate scans, thus minimizing the wasted time and money on the part of the patient, department, and medical insurers.

**Figure 8:**
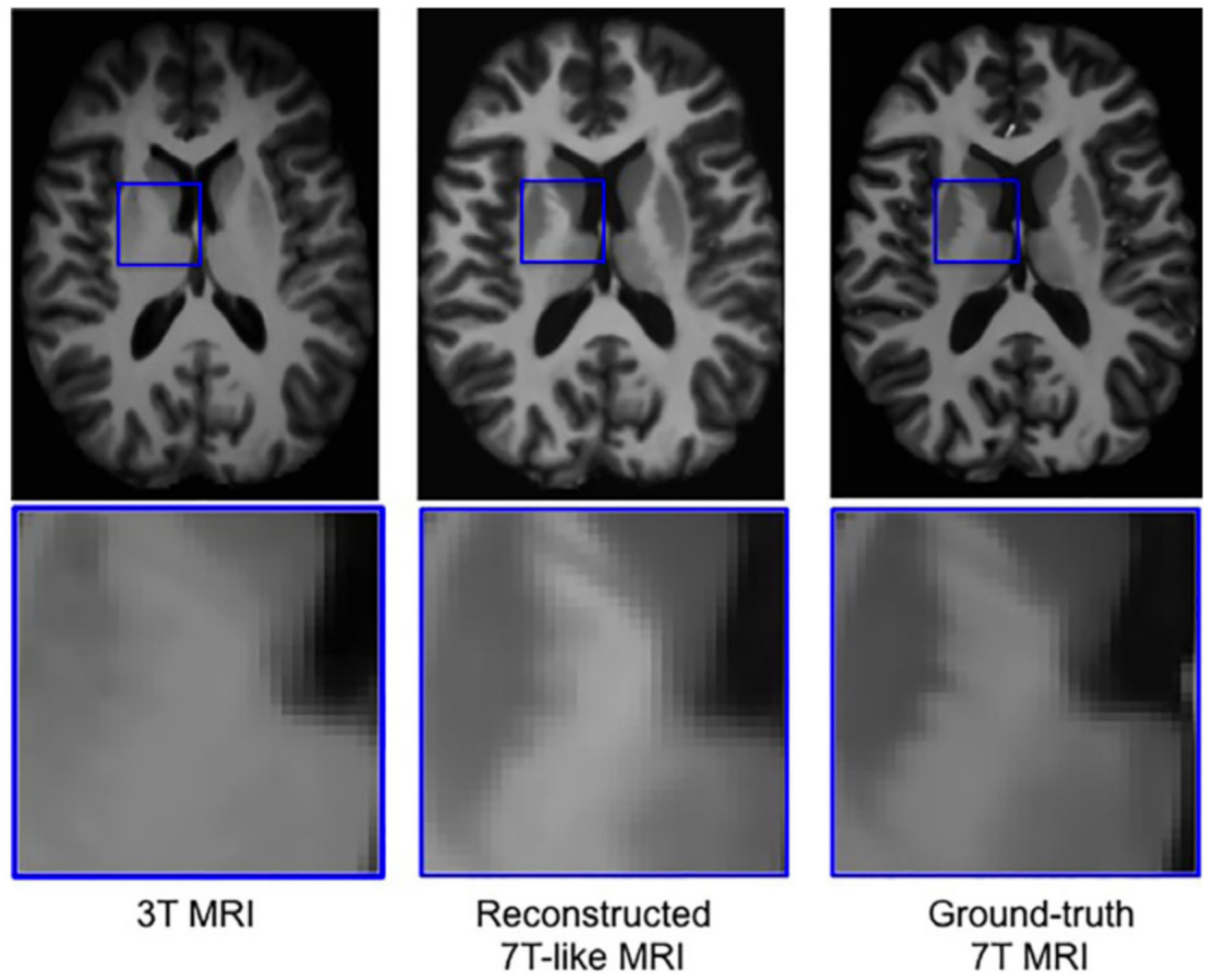
Example of a neural network trained on sets of 3T and 7T images, which is able to predict artificially upscaled 7T resolution images from actually acquired 3T input images (34). Reprinted from Bahrami et al. (46) with permission from John Wiley and Sons.

**Figure 9:**
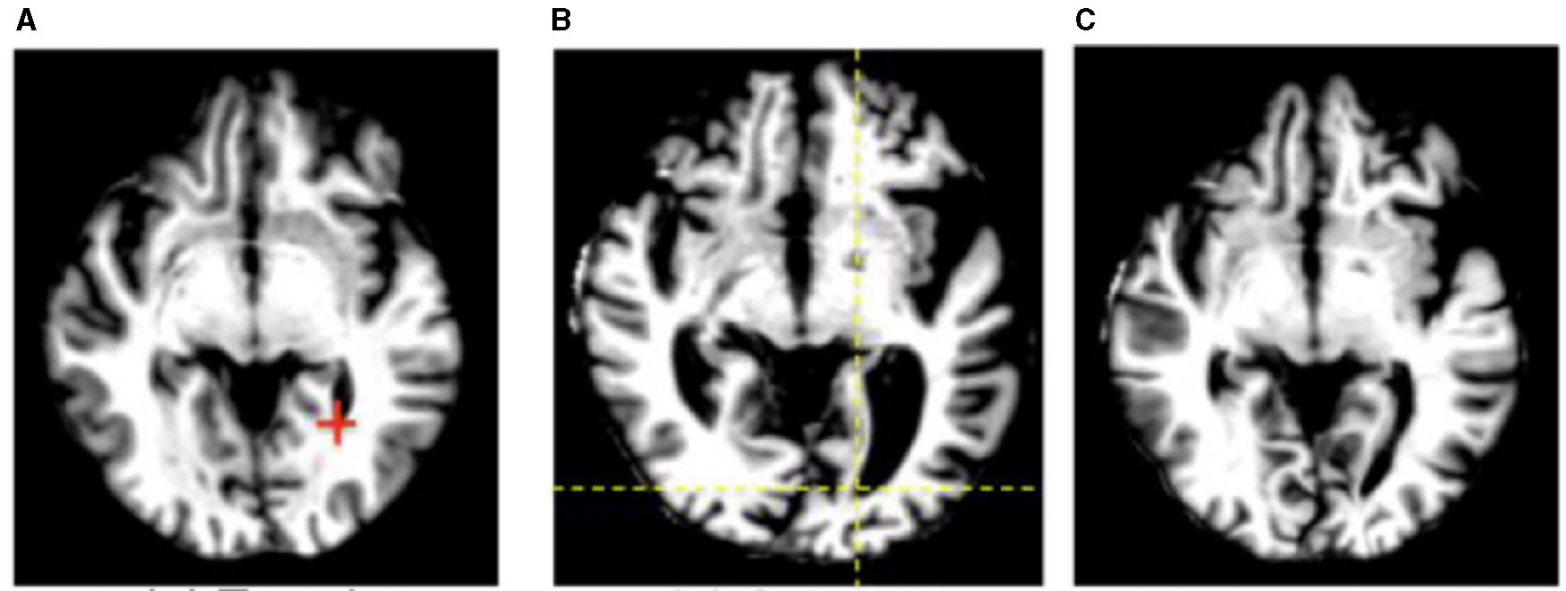
An example of automated image registration, in which a neural network is trained to identify landmarks in an image so that an input DICOM data array can be manipulated such that the way in which it is sliced matches a desired template slice, e.g. the same slice from a prior study (30). Reprinted from Wu et al. (42) with permission under the terms of the Creative Commons Attribution License, which permits unrestricted use, distribution, and reproduction in any medium, provided the original author and source are credited. **(A)** Template. **(B)** Subject. **(C)** Deformed subject.

**Figure 10:**
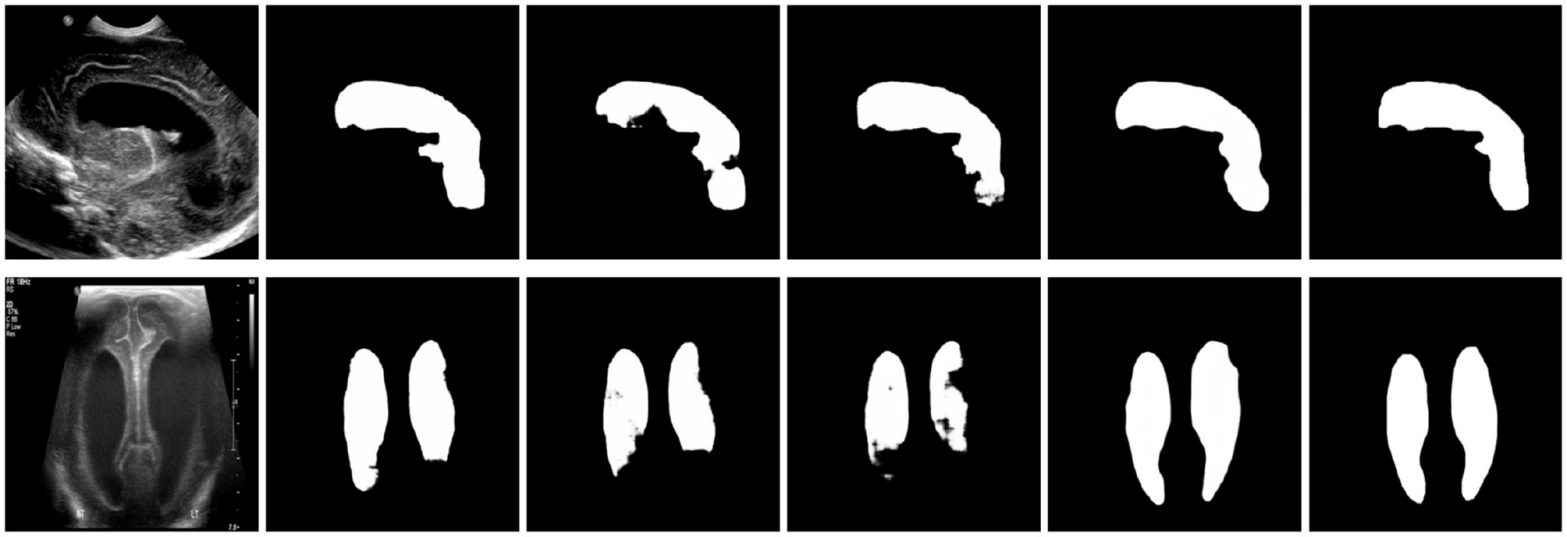
Example of US of the ventricles (left images) along with several neural network algorithms trained to automatically segment the ventricles (middle 4 images) compared to the ground truth manual segmentation (right images) 0.3 (6). Reprinted from Wang et al. (47) with permission under the terms of the Creative Commons Attribution License, which permits unrestricted use, distribution, and reproduction in any medium, provided the original author and source are credited.

One issue that is seen throughout all types of imaging is standardization. Significant differences in scanning equipment from different manufacturers, available sequences, institution-based protocols, and scanner settings can result in a large amount of variance in the imaging that results, and can cause difficulties for radiologists at different institutions to interpret the other institution's imaging. Even within a single institution, or for a single patient scanned serially, variability in which scanner is used or how the patient is positioned within the scanner can make direct comparisons challenging. There are a variety of methods in which AI can assist in such tasks, including the creation of augmented scans that “average” the look of similar sequences acquired from a variety of different magnets, artificially upscaling the resolution of a scan by interpolating data acquired at lower field strengths to mimic the appearance of high field strength imaging, and automatically rotating and scaling the DICOM data matrices such that a patients anatomy appears in the same position as on prior films, or automated image registration (4, 31, 42–46) (Figures 8, 9). These techniques allow for more uniform interpretation and comparison of imaging, maximizing the quality potentially lost through inevitable technical factors. Examples of the latter technique have already been deployed in pediatric neuroradiology in attempting to standardize head positioning when evaluating ventricular size, as well as in obtaining fetal biometrics, where the patients are notoriously prone to shifting position while being scanned (47–50) (Figure 10).

### Automating Labor-Intensive Tasks

**Figure 11:**
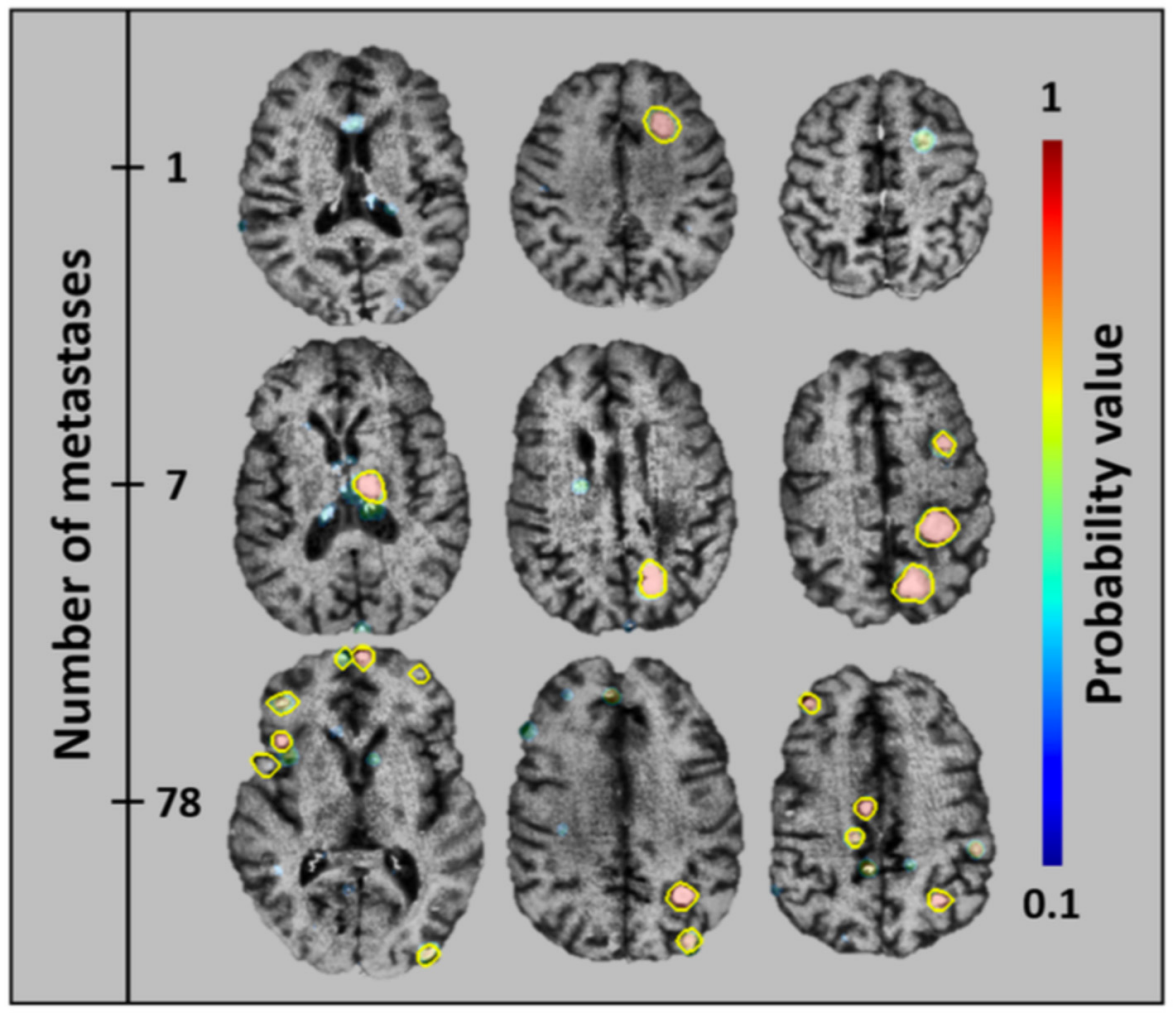
An algorithm that has been trained to detect metastatic brain lesions, which is also able to report the likelihood that the detected lesion is a genuine lesion. This algorithm is capable of reporting quantitative volumes of metastatic disease in less than a minute, well beyond the abilities of human radiologists (38). Reprinted from Grøvik et al. (51) with permission from John Wiley and Sons.

Another developing application of AI within adult neuroimaging is the routine monitoring for metastatic lesions within the brain. This task is often considered tedious by neuroradiologists given its time-consuming nature while at the same time requiring strict focus and consistent search pattern, lest a tiny lesion be missed. These features make this an ideal undertaking for automation, where any new lesions could be quickly detected and verified and changes in existing lesions can automatically be quantified. This task is already being undertaken in the adult world, where metastatic disease is a common entity, and regular follow up imaging is necessary to evaluate for progression (Figure 11) (51). With a few exceptions, metastatic disease is much less common in the pediatric population than it is in adults, however there are other diseases endemic to children such as NF1, VHL, and TSC as well as spinal metastases from medulloblastoma and other brain tumors that require a very similar technique in order to monitor for progression or recurrence. In fact, for some of these entities, such as serial evaluation of NF and TSC lesions or residual tumor following resection, automated detection could allow for quantitative volumetric analysis of such lesions. Having such data could potentially provide concrete quantitative guidance for clinical management of these patients.

### Bridging Knowledge and Skill Gaps in Radiology

One of the most important roles that AI could play in the field of pediatric neuroradiology is in assisting non-experts in interpretation. Pediatric radiology is a distinct subspecialty that can be very difficult and intimidating to radiologists who do not encounter it on a routine basis. Even normal anatomy can appear bizarre to those who are not familiar with it and can lead to a lack of confidence in interpretation. In settings where pediatric subspecialists are not available and/or a pediatric patient cannot be transferred to a such an institution, it is often necessary for general radiologists to interpret pediatric imaging themselves, especially given the perception of staffing shortages and high volumes that pediatric radiologists have historically reported (52, 53). An algorithm that is trained for pediatric interpretation could be immensely helpful in triaging such patients as emergent or likely normal or giving the interpreter enough assistance to make a preliminary diagnosis with some level of confidence. In this way AI could play a role in bridging the gap of knowledge between adult and pediatric radiologists.

In underserved communities and in the developing world, there has historically been a serious deficiency in specialized medicine of all types, particularly in pediatrics (52, 53). An AI system could be immensely beneficial in providing much-needed assistance to general radiologists serving such populations.

### Challenges in Pediatric Neuroradiology

Pediatric neuroradiology presents a unique set of challenges to the radiologist that do not exist in adult neuroradiology which may, at least in part, explain why AI has not become as well established as it has in the adult world. Perhaps the most likely reason for this disparity is that most research being conducted in adult neuroradiology focuses on relatively common entities such as stroke, trauma, and metastases that are much less prevalent in the pediatric patient population (54). This can potentially hinder research for a few reasons.

### Limited Scope of Practice

The development of algorithms is often focused on the most prevalent disease entities so that the resulting success will have the most impact and relevance. In their current state, deep learning models generally have a very narrow scope and tend to accomplish a single very specific task, such as segmenting a tumor or finding and quantifying metastatic or demyelinating lesions (51, 55). Because the development of an effective algorithm requires such a large amount of computational, financial, and time resources, the tasks that they are designed to focus on are generally the most common and prevalent conditions, and thus will be able to have the most clinical utility. Given the rarity and diversity of many pediatric conditions, there is typically a much smaller impact achievable.

## Limited Availability of Pediatric Data

As AI requires enormous amounts of data both normal and positive cases in order to appropriately train a network, both high prevalence of a disease as well as standardized imaging protocols are a necessity in order to develop an adequately large enough database of cases. The rarity and diversity of pediatric neurological entities makes it difficult to develop such a database in many cases, and such networks are often only able to be trained on datasets of a few hundred to a few thousand; for comparison, the prototypical ImageNet training dataset currently contains over 14 million annotated images, and the CheXNet chest X-ray dataset contains over 100,000 annotated frontal chest radiographs (9).

Nevertheless, as more de-identified data has become available and interest in deep learning has grown, many open source datasets have begun to become available on which researchers can train their algorithms. While these are still relatively rare in pediatrics, datasets are actively being constructed for more common pediatric entities, such as a collection of MRIs obtained from patients with proven hypoxic-ischemic encephalopathy (HIE). It is the intention that this database be used to develop MR biomarkers from an AI algorithm that can aid in lesion detection and outcome prediction (56).

To make matters even worse, the pediatric brain is distinct from the adult brain in that it is continuously in development, with dramatic changes in tissue signal occurring as myelination progresses (57). Because of this, what is normal for a 6 month old brain is completely different than what one would expect for that of a 2 year old. The variability in the cases that a neural network is trained on makes it challenging to establish a well-defined ground truth for the algorithm to compare against.

This is a challenge that is by no means isolated to neuroradiology, as the entire body of infants is rapidly evolving. In pediatric MSK imaging, for example, large databases have been collected and used for many years in order to monitor the growth of bones of the hand so that patient “bone age” can be estimated. The availability of such a database as well as the somewhat tedious nature of this task makes it appear to be tailor made for automation. The RSNA released a dataset of more than 14,000 such hand radiographs as part of a challenge to develop the most accurate ML algorithm for predicting bone age, resulting in over 100 submitted algorithms with a winning average estimation of 4.2 months within the actual patient age (58–60). Additional models have since been developed using radiographs of the index finger with mean error of approximately 5 months and even MRIs of the knee with a mean error of 9–12 months (61, 62). The state of cerebral myelination is a useful indicator of neural maturity and neonatal development and can aid in determining between term and pre-term brains (57, 63). Just as with bone age estimation, an algorithm that can accurately determine brain age would be a very profitable deployment of machine learning in terms of treatment and outcomes. While there has been some success with developing an algorithm for determining brain age in adults, there is currently no large annotated MRI dataset available, normal or otherwise, that can be used to train a neural network (64).

### Limited Availability of Skilled Practitioners

Research into machine learning within the field of pediatric neuroradiology is likely limited simply by the relatively small number of pediatric neuroimagers available to focus on such research. According to the 2018 ACR Commission on Human Resources Workforce Survey, only 3.8% of board certified radiologists (on the order of approximately 1000 radiologists) practicing primarily pediatric radiology in the US, with only a very small percentage of these practicing primarily pediatric neuroradiology. This is far less than primary adult neuroradiologists (12.4%) and many more AI researchers (5). The majority of available publications on the subject are submitted by computer science and general AI researchers, rather than radiologists. This a common trend seen in AI research, and one could accurately describe the field of machine learning in medical imaging as more of a sub-specialization of artificial intelligence rather than a developing technique in radiology. This being the case, the smaller number of practitioners within pediatric neuroradiology makes it a relatively smaller voice in the fervor of medical AI research.

### Strategies to Tackle Challenges

So where does this leave the pediatric neuroradiologist within the field of AI? At first glance, the challenges facing the deployment and widespread use of AI in pediatric neuroradiology may seem formidable and could prevent the widespread adoption of the technology in the field. Fortunately, there are several potential strategies that could help deal with these apparent roadblocks and enable the field to benefit from the technology.

One technique that could allow many pre-existing algorithms that have been developed for adult applications to be applied directly to the pediatric population is that of transfer learning. Transfer learning refers to the ability of a network that has been trained on a separate dataset being able to successfully run on a different, though related, dataset (65, 66). An example of this would be a speech-to-text algorithm that was trained on English words to attempt to transcribe Spanish words. Though the data it was trained on is in a completely differently language and there will be few words that appear in both sets, the sounds that make up the languages are similar enough that the algorithm will likely be able to complete the task with some measure of success (67). Similarly, the appearance of an adult and pediatric chest X-ray may be significantly different from the perspective of a human radiologist, they are much more similar than an adult chest X-ray and a still life of fruit, for example, and a neural network will generally be able transfer some of its diagnostic skills across this gap (1). This means that any algorithm developed for an adult application can be applied, with some success, to its pediatric counterpart, assuming the manifestation has similar imaging characteristics.

Transfer learning can also be applied to overcome the insufficiency of datasets in pediatric neuroimaging. By expanding the training dataset for a pediatric based algorithm to be able to include adult imaging of analogous entities, the network will become more robust, though with a potential slight decrease in specificity for the features that are unique in the pediatric version of the entity. Another way to tackle the issue of underpowered pediatric datasets is to ensure that the dataset being used is as “clean” as possible (40). Since the neural network will be optimized according to its training dataset, any errors within the dataset can deteriorate the algorithm's accuracy. A meticulously edited dataset, where each entry has been properly annotated and calibrated has been shown to significantly decrease the size of the dataset required to train neural networks in order to obtain adequate results for certain tasks, reducing the size of datasets from tens of thousands to only hundreds or even dozens (40). Unfortunately, while uniformity in datasets may reduce the number of examples necessary for training a network, it typically comes at the cost of reducing the robustness of the algorithm, and arguments have been made that a certain amount of “contamination” of datasets is a necessary component of training a network that is robust enough to practically function in real world situations, which are rarely if ever perfectly uniform (11).

Ironically, if one wants to develop a dataset that is as uniform as possible, one way to achieve this is to use AI. There have been multiple attempts to develop algorithms (with and without deep learning) that are able to take an arbitrary image and manipulate the DICOM data in such a way that it is displayed in a standardized orientation and window/level. This process not only makes it more aesthetically pleasing for human radiologists to read studies, but also makes it much easier to train networks and may also be a necessary preprocessing step before some algorithms can perform tasks such as ventricle comparisons, automated segmentation, and determination of lesion progression.

## Conclusion

The promise that AI holds for fundamentally transforming radiology cannot be overstated. Notably, there are features of pediatric neuroradiology that could benefit from AI that are unique from other subspecialties. Improvements in image acquisition speed, dose reduction, motion artifact improvement, and interpretation assistance are important in all subfields of radiology, but are particularly well-suited for pediatric imaging. As research in the subject of machine learning and artificial intelligence continues to progress, radiologists will see an increasing amount of application into our daily practice. Within the coming years, the few examples from the developing AI toolset that has been discussed in this paper will become as commonplace and indispensable to modern radiology as PACS or dictation software is today.

## Author Contributions

DM: topic creation, manuscript preparation, editing, and revising. ET, BK, and KY: topic creation and editing. VY: topic creation, manuscript preparation, and editing. All authors contributed to the article and approved the submitted version.

## Conflict of Interest

The authors declare that the research was conducted in the absence of any commercial or financial relationships that could be construed as a potential conflict of interest.

## Publisher's Note

All claims expressed in this article are solely those of the authors and do not necessarily represent those of their affiliated organizations, or those of the publisher, the editors and the reviewers. Any product that may be evaluated in this article, or claim that may be made by its manufacturer, is not guaranteed or endorsed by the publisher.
